# The Association Between Community Participation and Loneliness Among Patients in Rural Community Hospitals: A Cross-Sectional Study

**DOI:** 10.7759/cureus.56501

**Published:** 2024-03-19

**Authors:** Ryuichi Ohta, Toshihiro Yakabe, Hiroshi Adachi, Chiaki Sano

**Affiliations:** 1 Community Care, Unnan City Hospital, Unnan, JPN; 2 Family Medicine, Unnan City Hospital, Unnan, JPN; 3 Community Medicine Management, Shimane University Faculty of Medicine, Izumo, JPN

**Keywords:** community networks, body mass index, aged, social participation, rural population, loneliness

## Abstract

Introduction

Loneliness among adults is a critical public health issue, particularly in rural areas where social isolation can be more pronounced. Understanding the factors that influence loneliness can guide the development of effective interventions. This study explores the impact of demographic, health-related, and social participation factors on loneliness among rural Japanese adults, focusing on the role of community participation.

Method

This cross-sectional study was conducted with rural Japanese adults who regularly visited rural community hospitals. Data were collected on participants' demographic characteristics, health status, and social participation and analyzed using a multivariate logistic regression model to identify factors associated with higher levels of loneliness. The covariates included age, sex, body mass index (BMI), chronic health conditions, and community participation.

Results

The study found that community participation had a significant negative association with loneliness, with an odds ratio (OR) of 0.46 (p < 0.01), indicating that individuals engaged in community activities were substantially less likely to experience higher levels of loneliness. Higher BMI was associated with lower odds of loneliness (OR = 0.93, p < 0.02), suggesting a protective effect against loneliness.

Conclusion

The findings highlight the paramount importance of community engagement in mitigating loneliness among rural Japanese adults. The inverse relationship between BMI and loneliness suggests that BMI and social participation influence loneliness. These insights underscore the need for comprehensive interventions that promote community participation and address the multifaceted nature of loneliness. Future research should further explore the mechanisms through which community engagement and BMI impact loneliness to develop targeted strategies for improving the well-being of rural adults.

## Introduction

In recent years, the intricate connection between social isolation and individual health outcomes, such as quality of life and chronic diseases, has garnered significant attention from researchers and healthcare professionals worldwide [1,2]. This growing body of research underscores the critical role of socioeconomic factors in influencing overall health, particularly in vulnerable populations such as rural adults [3,4]. Among the various dimensions of social interaction, community participation emerges as a pivotal element, potentially serving as a buffer against the negative health impacts of isolation [5,6].

The adverse effects of social isolation on health are multifaceted, encompassing psychological stress, limited access to healthcare resources, and a lack of emotional and social support [7,8]. These factors collectively contribute to the deterioration of an individual's health condition, highlighting the importance of social ties and community engagement in promoting health and well-being [7,8]. This relationship is especially pronounced in rural areas, where adults often face more significant challenges in accessing community resources and engaging in social activities, thereby exacerbating their risk of isolation and its associated health consequences [9,10].

The hypothesis that community participation can mitigate the effects of social isolation and improve health outcomes forms the basis of this research. By fostering a sense of belonging and providing opportunities for social engagement, community involvement is posited to enhance individuals' mental and physical health, potentially leading to lower levels of loneliness and improved management of chronic conditions [3,4,11]. This is particularly relevant in the context of aging populations in rural settings, where social networks may be more limited and the impact of isolation more acute [12,13].

Despite the intuitive link between community participation and health, empirical evidence exploring this association, especially in chronic disease management and loneliness, remains sparse [14]. The current research aims to fill this gap by examining whether active engagement in community activities is associated with lower levels of loneliness and better health outcomes among the elderly in rural areas. Given the complex interplay between social isolation, loneliness, and health, this study seeks to provide a comprehensive understanding of how community participation can serve as a key intervention strategy in reducing loneliness and improving the quality of life of rural adults.

This cross-sectional study utilized quantitative methods to assess the level of community participation among adults in rural areas and its relationship with risk factors of loneliness. By identifying the extent to which community engagement influences these outcomes, the research aims to contribute to developing targeted interventions designed to promote social inclusion and improve health outcomes. The findings of this study could have significant implications for public health strategies and policies aimed at reducing loneliness, particularly in underserved rural communities.

## Materials and methods

This cross-sectional study was performed with rural citizens who regularly visited a rural Japanese community hospital to clarify the association between community participation and feelings of loneliness.

Setting

Unnan City, one of the most rural cities in Japan, is located in the southeastern part of Shimane Prefecture. In 2020, the total population of Unnan was 37,638 (18,145 men and 19,492 women), 39% of which were aged over 65 years. The older population is expected to reach 50% by 2025. There are 16 clinics, 12 home care stations, three visiting nurse stations, and one public hospital (Unnan City Hospital) in Unnan City [15]. Unnan City has 16 clinics, three visiting nurse stations, and 12 home care stations. Care managers work individually or belong to home care stations. They engage with home care patients, their families, and other medical and care professionals to manage patients' care contents and determine the need for professional help. Home care workers belong to home care stations and support home care patients' lives through physical care, assisted living, and transportation services [16].

Participants

All patients older than 40 who regularly visited Unnan City Hospital were included between September 1, 2023, and November 31, 2023. To collect the data, data on patients who regularly visited the hospital for their chronic diseases or annual health checks were extracted for electrical medical records for analysis. In addition, to measure the degree of loneliness and participation in community activities, the questionnaire of the Japanese version of the three-item University of California, Los Angeles (UCLA) Loneliness Scale was sent to the participants [17].

Measurements

Primary Outcome

The degree of loneliness was assessed using the Japanese version of the three-item UCLA Loneliness Scale among community-dwelling older adults (score range: 3-9). The scale consists of three items: Item 1 (companionship), How often do you feel you lack companionship? (Scale of 1-3); Item 2 (leftover), How often do you feel left out? (Scale of 1-3); and Item 3 (Isolated), How often do you feel isolated from others? (Scale of 1-3). We calculated the loneliness scale by summing the score for each item [17].

Independent Variable

We used a questionnaire to collect data about community participation. Regarding community activities, participants were asked about their concrete participation in them voluntarily. The question was, “Do you participate regularly in community activities in your communities, such as salons, festivals, educational and cultural activities, and exercise programs?” The participants answered the question with a yes or no [11].

Covariate

The background data of the participants were collected from the electronic patient records of Unnan City Hospital [16]. The following patient data were collected: age, sex, body mass index for nutritional assessment, serum creatinine level (mg/dL), estimated glomerular filtration rate (mL/min/1.73 m^2^) for renal function assessment, and Charlson Comorbidity Index (CCI) for the assessment of the severity of comorbidities (heart failure, myocardial infarction, asthma, chronic obstructive pulmonary disease, kidney disease, liver disease, diabetes mellitus, brain infarction, brain hemorrhage, hemiplegia, connective tissue diseases, dementia, and cancer) [18]. The laboratory data were from the participants' last visits to the hospital for their chronic diseases or annual health checks.

Statistical analysis

Student’s t-test was used to analyze parametric data, whereas the Mann-Whitney U test was used to analyze nonparametric data. Numerical variables were dichotomized based on the information of the median of the variable: the score of loneliness, ≥4 (higher loneliness) and <4 (lower loneliness) because the mean and median of the variable were similar (average, 4.17; standard deviation (SD), 1.42; median, 4; interquartile range, 2). A univariate regression model was used to determine whether community participation was associated with independent variables and covariates. A multivariate logistic regression analysis explored the association between community participation and higher loneliness. Only variables correlated with community participation in the univariate regression analysis (p-value, around 0.1 and <0.1) were considered in the multivariate logistic model. Participants with missing data were excluded. Statistical significance was set at p < 0.05. All statistical analyses were performed using EZR (Saitama Medical Center, Jichi Medical University, Saitama, Japan), which is a graphical user interface for R (The R Foundation, Vienna, Austria) [19].

Ethical considerations

The hospital was assured of the anonymity and confidentiality of the patient information used in this study. Information related to this study was posted on the hospital website without disclosing any patient details. The contact information of the hospital representative was also listed on the website to ensure that any questions regarding this study were addressed. All participants were informed of the purpose of this study and provided informed consent by completing the content form. The Unnan City Hospital Clinical Ethics Committee approved the study protocol (approval code: 20230010).

## Results

Participants' demographic

Between September 1, 2023, and November 31, 2023, 1024 patients were regularly followed by the general medicine department. The questionnaires were sent to all the patients. In total, 647 participants who answered the questionnaires were included in this study. The research investigated multiple health and demographic factors among a cohort of 647 participants, distinguishing between those who engage in community participation and those who do not. The average age of participants was 71.26 years, with a slightly higher mean age observed in the community participation group (73.00 years) compared to the non-community participation group (70.41 years), a statistically significant difference (p = 0.011). Gender distribution was nearly equal across both groups, with males comprising approximately 97 (46%) participants in the group with community participation, and no significant difference in gender distribution was noted (p = 0.933).

Regarding health indicators, the mean albumin level was 4.10, with a slightly higher level in the community participation group, though this difference was not statistically significant (p = 0.194). Similarly, other physical measurements, such as height, body weight, and BMI, showed no significant differences between the two groups, indicating that community participation does not have a marked impact on these physical health parameters.

The measures related to social well-being and mental health, such as the loneliness scale, companionship, and feelings of isolation, showed significant differences. Participants engaged in community activities reported lower loneliness scores and higher companionship levels than those not participating, suggesting a positive impact of community engagement on these aspects. The loneliness scale highlighted a significant disparity, with a mean score of 3.73 in the community participation group versus 4.38 in the non-participation group (p < 0.001) (Table 1).

**Table 1:** Demographics of the participant BMI, body mass index; CCI, Charlson Comorbidity Index; CKD, chronic kidney diseases; COPD, chronic obstructive pulmonary diseases; eGFR, estimated glomerular filtration rate; MI, myocardial infarction; SD, standard deviation

Factor	Total	Community participation	Non-community participation	p-value
N	647	211	436	
Age, mean (SD)	71.26 (12.18)	73.00 (10.96)	70.41 (12.65)	0.011
Male sex (%)	299 (46.3)	97 (46.0)	202 (46.4)	0.933
Albumin, mean (SD)	4.10 (0.41)	4.13 (0.32)	4.08 (0.44)	0.194
Height (cm), mean (SD)	158.63 (8.60)	158.25 (8.97)	158.81 (8.42)	0.433
Body weight (kg), mean (SD)	58.28 (12.79)	57.98 (12.80)	58.43 (12.80)	0.676
BMI, mean (SD)	23.00 (3.81)	22.96 (3.61)	23.02 (3.91)	0.858
eGFR, mean (SD)	63.93 (15.49)	62.67 (14.93)	64.54 (15.73)	0.151
Hemoglobin, mean (SD)	13.28 (1.44)	13.24 (1.42)	13.30 (1.45)	0.623
Hemoglobin A1c, mean (SD)	5.77 (0.56)	5.78 (0.56)	5.77 (0.55)	0.79
LDL, mean (SD)	106.26 (23.85)	106.40 (21.90)	106.19 (24.76)	0.915
Loneliness scale, mean (SD)	4.17 (1.42)	3.73 (1.13)	4.38 (1.50)	<0.001
Companionship, mean (SD)	1.54 (0.63)	1.36 (0.54)	1.63 (0.65)	<0.001
Isolated, mean (SD)	1.30 (0.51)	1.17 (0.39)	1.36 (0.55)	<0.001
Leftover, mean (SD)	1.33 (0.52)	1.19 (0.42)	1.40 (0.55)	<0.001
Higher loneliness (%)	337 (52.1)	82 (38.9)	255 (58.5)	<0.001
CCI ≥ 5 (%)	218 (33.7)	79 (37.4)	139 (31.9)	0.183
CCI (%)				
0	20 (3.1)	5 (2.4)	15 (3.4)	0.541
1	57 (8.8)	15 (7.1)	42 (9.6)	
2	82 (12.7)	24 (11.4)	58 (13.3)	
3	142 (21.9)	43 (20.4)	99 (22.7)	
4	128 (19.8)	45 (21.3)	83 (19.0)	
5	107 (16.5)	46 (21.8)	61 (14.0)	
6	64 (9.9)	24 (11.4)	40 (9.2)	
7	34 (5.3)	9 (4.3)	25 (5.7)	
8	10 (1.5)	0 (0.0)	10 (2.3)	
9	3 (0.5)	0 (0.0)	3 (0.7)	
Heart failure (%)	55 (8.5)	12 (5.7)	43 (9.9)	0.097
MI (%)	5 (0.8)	0 (0.0)	5 (1.1)	0.179
Asthma (%)	43 (6.6)	8 (3.8)	35 (8.0)	0.044
Peptic ulcer (%)	55 (8.5)	22 (10.4)	33 (7.6)	0.231
Kidney disease (%)	168 (26.0)	62 (29.4)	106 (24.3)	0.181
Liver disease (%)	52 (8.0)	13 (6.2)	39 (8.9)	0.28
COPD (%)	38 (5.9)	7 (3.3)	31 (7.1)	0.073
DM (%)	130 (20.1)	42 (20.0)	88 (20.2)	1
Brain infarction (%)	51 (7.9)	12 (5.7)	39 (8.9)	0.164
Brain hemorrhage (%)	13 (2.0)	3 (1.4)	10 (2.3)	0.562
Connective tissue disease (%)	85 (13.1)	27 (12.8)	58 (13.3)	0.902
Dementia (%)	12 (1.9)	2 (0.9)	10 (2.3)	0.354
Cancer (%)	69 (10.7)	23 (10.9)	46 (10.6)	0.893
Hypertension (%)	428 (66.2)	149 (70.6)	279 (64.0)	0.111
Dyslipidemia (%)	388 (60.0)	132 (62.6)	256 (58.7)	0.392

The multivariate logistic regression model

The multivariate logistic regression model explored the impact of various factors on the likelihood of higher loneliness. Age, with an odds ratio (OR) of 0.99, and male sex, with an OR of 1.06, had minimal influence on higher loneliness. In contrast, higher BMI was associated with decreased odds of higher loneliness (OR = 0.93, p < 0.02). The presence of community participation was associated with lower loneliness (0.46, p < 0.01), suggesting that individuals engaged in community activities are significantly less likely to have higher loneliness (Table 2).

**Table 2:** The multivariate logistic regression model BMI, body mass index; CCI, Charlson Comorbidity Index; COPD, chronic obstructive pulmonary diseases; CI, confidential interval

Factor	Odds ratio	95%CI	P value
Age	0.99	0.98-1.01	0.34
CCI≧5	0.82	0.52-1.27	0.37
Male sex	1.06	0.75-1.49	0.75
Community participation	0.46	0.33-0.66	<0.01
BMI	0.93	0.89-0.97	<0.02
Serum albumin	1.05	0.70-1.59	0.8
Asthma	1.79	0.91-3.55	0.094
COPD	0.72	0.35-1.47	0.36
Heart failure	1.42	0.76-2.68	0.27

## Discussion

The intricate relationship between loneliness, community participation, and BMI among rural Japanese adults provides a fascinating lens through which we can understand the complex interplay of social and personal determinants of well-being. This discussion delves into the findings from the multivariate logistic regression model, emphasizing the influence of community participation and BMI on loneliness.

The standout result from this study is the potent negative association between community participation and higher levels of loneliness, with an odds ratio (OR) of 0.46 (p < 0.01). This finding underscores the critical role that social engagement plays in mitigating feelings of loneliness among the elderly [20]. Engaging in community activities gives rural adults a sense of belonging, purpose, and social support, vital for their mental and emotional well-being [21,22]. The data strongly suggest that initiatives aimed at increasing community involvement among the elderly could be a key strategy in reducing loneliness, which is a growing concern in aging societies [23].

Another intriguing finding is the inverse relationship between higher BMI and the odds of experiencing higher loneliness (OR = 0.93, p < 0.02). While obesity and higher BMI are often associated with various health risks, this result might indicate that among rural Japanese adults, those with higher BMI may have more social interactions or engagements that protect against loneliness [24,25]. This could be due to cultural differences in perceptions of body image or the role of food and communal eating in fostering social bonds [26]. However, the exact mechanisms behind this relationship warrant further investigation into how physical health factors intersect with social well-being.

The analysis also revealed that age and male sex did not influence higher loneliness, with odds ratios of 0.99 and 1.06, respectively. These findings challenge some commonly held assumptions about the risk factors for loneliness, suggesting that in this population, demographic factors like age and sex are not significant determinants of loneliness [24,25]. This highlights the importance of looking beyond demographic variables to understand the multifaceted nature of loneliness among rural adults.

The insights from this study have significant implications for policy and practice. They indicate that interventions to reduce loneliness among rural adults should prioritize fostering community connections and participation [27,28]. This could involve developing community centers, social clubs, or volunteering programs specifically designed to meet the interests and needs of the elderly population [29,30]. Additionally, the relationship between BMI and loneliness suggests that interventions should also consider the physical well-being of rural adults, not promoting thinner body styles that can support both physical health and social engagement.

While illuminating the positive effects of community participation on loneliness among rural Japanese adults, this study has several limitations. First, its cross-sectional design precludes causal inference, allowing longitudinal studies to explore temporal relationships. Additionally, the reliance on self-reported measures introduces potential bias, necessitating objective assessments in future research. The study's geographic specificity to rural Japan may also limit the generalizability of findings to other cultural or urban contexts. Addressing these limitations would enhance our understanding of the complex dynamics between community engagement and loneliness in rural adults.

## Conclusions

This study provides empirical evidence supporting the beneficial effects of community participation on reducing feelings of loneliness among adults in rural Japan. By demonstrating that social engagement can serve as a key factor in enhancing this population's mental and emotional well-being, our research highlights the importance of fostering community connections as part of a holistic approach to health and well-being. Future efforts should focus on developing and implementing community-based interventions that are accessible and relevant to adults, particularly in rural settings, to combat loneliness and promote a more inclusive and supportive social environment.
